# Effects of Fibrafid Supplementation in Standard and Nutrient-Reduced Diets on Growth Performance, Production Efficiency, Carcass Traits, Meat Quality, and Intestinal Morphology in Broilers

**DOI:** 10.3390/vetsci13080792

**Published:** 2026-08-08

**Authors:** Maged A. Al-Garadi, Rashed A. Alhotan, Elsayed O. Hussein, Mohammed M. Qaid, Gamaleldin M. Suliman, Esam H. Fazea, Mohammed A. Al-Badwi, Isiaka O. Olarinre

**Affiliations:** Department of Animal Production, College of Food and Agriculture Sciences, King Saud University, Riyadh 11451, Saudi Arabia; ralhotan@ksu.edu.sa (R.A.A.); shessin@ksu.edu.sa (E.O.H.); mqaid@ksu.edu.sa (M.M.Q.); gsuliman@ksu.edu.sa (G.M.S.); 441106617@student.ksu.edu.sa (E.H.F.); malbadwi@ksu.edu.sa (M.A.A.-B.); 446110183@student.ksu.edu.sa (I.O.O.)

**Keywords:** amino acid density, broilers, Fibrafid, growth performance, intestinal histomorphology, meat quality, metabolizable energy, nutrient-reduced diet, plant-based feed additive

## Abstract

This study evaluated the effects of Fibrafid, a plant-based functional feed additive, on growth performance, carcass traits, meat quality, and intestinal histomorphometry of broilers fed standard or moderately nutrient-reduced diets. A total of 432 one-day-old Ross 308 chicks (initial body weight, 44.9 ± 0.30 g) were randomly allocated to a 2 × 3 factorial arrangement comprising two dietary nutrient densities (standard or diets with 1.5% lower metabolizable energy and 5% lower digestible amino acid density) and three Fibrafid inclusion levels (0, 1.5, or 2.5 kg/ton). Each treatment comprised six replicates of 12 birds (six males and six females) and was evaluated from day 1 to 24. Dietary nutrient reduction affected growth performance and breast meat quality pH and ultimate lightness values, whereas Fibrafid supplementation, particularly at 2.5 kg/ton, improved overall gain by 5.3%, grower-phase feed conversion ratio by 4.0%, EPEF by 8.08%, relative thymus weight by 64.8%, while reducing abdominal fat deposition by 27.1% relative to the unsupplemented control (*p* < 0.05). Several breast meat quality traits were also improved, including greater water-holding capacity and myofibril fragmentation index and lower drip and cooking losses (*p* < 0.05). Jejunal morphology was enhanced, as indicated by increased villus dimensions and goblet cell density (*p* < 0.05). Significant Diet × Fibrafid interactions were observed for grower performance, EPEF, overall feed intake, and several intestinal morphological variables (*p* < 0.05). In conclusion, Fibrafid supplementation, particularly at 2.5 kg/ton, was associated with improved growth performance, EPEF, selected carcass and breast meat quality traits, and jejunal morphology during the first 24 days of age. These findings suggest that Fibrafid may be a promising natural feed additive for broiler diets formulated at standard or moderately reduced nutrient densities. However, further studies extending to market age and evaluating nutrient digestibility, intestinal microbiota, blood biochemical indices, and economic returns are warranted to clarify its mechanisms and practical value under commercial production conditions.

## Simple Summary

Feed cost and efficient nutrient use are major challenges in modern broiler production. Natural feed additives are increasingly used to support growth performance and intestinal health, but many phytogenic products primarily rely on essential oils or plant extracts with antioxidant and antimicrobial properties. Fibrafid differs by combining plant-derived fermentable fiber with organic acids, potentially providing both prebiotic and digestive-support functions. This study evaluated the effects of Fibrafid supplementation in broilers fed either standard diets or moderately nutrient-reduced diets with lower metabolizable energy and digestible amino acid density. Fibrafid supplementation, particularly at 2.5 kg/ton, improved body weight gain, feed efficiency, European production efficiency factor, carcass leanness, breast meat quality, and intestinal development. Importantly, Fibrafid supplementation was associated with maintaining productive performance under moderately nutrient-reduced dietary conditions. These results suggest that Fibrafid may be a promising natural feed additive for improve broiler productivity and supporting greater flexibility in feed formulation programs. Further research extending to market age and evaluating nutrient digestibility, intestinal microbiota, economic returns, and other underlying mechanisms is needed to confirm its practical value under commercial production conditions.

## 1. Introduction

Feed accounts for the largest proportion of poultry production costs, with dietary protein and energy sources representing the most expensive components of broiler diets [1,2,3,4,5]. At the same time, increasing pressure to improve production efficiency while reducing the environmental footprint of poultry farming has intensified interest in sustainable nutritional strategies [6,7]. Conventional corn–soybean meal-based diets are effective in supporting rapid growth; however, their reliance on high levels of protein-rich ingredients increases feed costs and contributes to nitrogen excretion, which may negatively affect environmental sustainability [8,9,10,11]. Consequently, improving nutrient-use efficiency and reducing dependence on costly dietary inputs have become important objectives in modern poultry nutrition, prompting the development of feeding strategies that maintain productive performance while minimizing economic and environmental costs [12,13].

Crude protein (CP) is central to poultry nutrition because it supplies the essential amino acids (AAs) required for growth. Although non-essential AAs can be synthesized from excess essential AAs and amino nitrogen [10,14], conventional commercial broiler diets are frequently formulated with crude protein levels above physiological requirements to provide formulation safety margins and ensure adequate amino acid supply under practical production conditions. Consequently, excess dietary protein may increase nitrogen excretion and associated environmental and metabolic burdens [8,15,16]. Although low-protein diets (LPRO) fortified with balanced digestible amino acid profile offer potential, they sometimes fail to maintain optimal performance [17]. Therefore, reducing dietary CP while maintaining an adequate balanced digestible amino acid profile has become an important nutritional strategy for minimizing nitrogen emissions while maintaining production performance [9,18,19,20,21]. Recent studies consistently indicate that moderate reductions in dietary CP (approximately 1.5–2.0%) can be implemented successfully when limiting digestible amino acids are precisely balanced, allowing broilers to maintain growth performance with minimal adverse effects on productivity [22,23,24,25,26]. In some cases, low-protein diets have also improved selected metabolic indicators and reduced nitrogen excretion, highlighting their potential contribution to more sustainable poultry production. Nevertheless, the magnitude and consistency of these responses vary among studies, reflecting differences in dietary formulation, amino acid balance, bird age, genetic background, and management conditions. Consequently, additional nutritional strategies are still needed to improve nutrient utilization and support performance under nutrient-reduced feeding programs.

Because dietary protein and energy together account for the largest proportion of feed cost and interact closely in regulating nutrient intake, growth, and feed efficiency, commercial feed formulations frequently optimize both nutrients simultaneously rather than independently. Energy and AAs are also the most expensive components of broiler diets. Modern broiler genotypes appear to require proportionally higher AA supply relative to metabolizable energy (ME) than earlier strains [27,28]. Consequently, the challenge is not only to reduce dietary CP but also to optimize the balance between amino acid density and dietary ME while maintaining productive performance. Increasing digestible amino acid density while moderately lowering ME has been associated with improvements in nutrient utilization, carcass yield, and breast meat quality [29]. Consistent with this approach, the latest Ross 308 Broiler Nutrition Specifications and Cobb Broiler Nutrition Guide recommend lower dietary ME concentrations while increasing digestible amino acid specifications to improve nutrient efficiency and production performance [30,31]. Moreover, moderate reductions in dietary ME (50–150 kcal/kg) can be implemented without major performance losses when diets are properly balanced [29,32]. Nevertheless, excessive reductions in dietary energy may compromise digestive efficiency by limiting the energy available for intestinal tissue maintenance, digestive enzyme synthesis, and active nutrient transport, particularly during periods of rapid intestinal development [33,34]. Such reductions may also alter digesta passage rate and nutrient digestibility, thereby reducing the efficiency of nutrient absorption when compensatory increases in feed intake are insufficient. Consequently, there is increasing interest in functional feed additives that support gastrointestinal function and nutrient utilization in low-protein and reduced-energy feeding programs. However, responses to functional feed additives in low-protein or reduced-energy diets have been inconsistent. Although functional fiber sources, probiotics, prebiotics, organic acids, enzymes, and phytogenic compounds have individually shown potential to improve nutrient utilization and gut health, particularly when birds are exposed to nutritional or environmental challenges [11,35,36,37,38,39,40,41,42], their efficacy varies considerably depending on plant species and cultivar, growing and harvest conditions, extraction and processing methods, the concentration and stability of bioactive compounds, dietary composition, and interactions with other feed ingredients, as well as differences in bird age, health status, and environmental conditions [43,44]. From a practical perspective, additives that improve nutrient efficiency may help maintain productivity while allowing more flexible feed formulation and partial reductions in costly protein and energy inputs [45,46,47]. Furthermore, most previous studies have evaluated these additives individually, with limited information available on multifunctional products that combine fermentable fiber and organic acids to simultaneously support intestinal health and nutrient utilization under nutrient-reduced feeding programs. Consequently, it remains unclear whether such multifunctional feed additives can effectively compensate for simultaneous reductions in dietary amino acid density and metabolizable energy while maintaining growth performance, carcass traits, meat quality, and intestinal development. Addressing this knowledge gap is important for developing sustainable nutritional strategies that reduce feed costs and nitrogen excretion without compromising broiler productivity.

To address this knowledge gap, Fibrafid was evaluated because it combines plant-derived fermentable fiber, organic acids, and readily fermentable substrates that have individually been associated with improvements in gastrointestinal function and nutrient utilization. Unlike conventional phytogenic additives, which primarily rely on bioactive plant extracts, or simple fiber sources that mainly provide structural carbohydrates, Fibrafid integrates fermentable fiber and acidifying components within a single product. Fermentable fiber has been reported to promote intestinal development and microbial fermentation, whereas organic acids help maintain a favorable gastrointestinal environment and improve nutrient digestion [43,44]. However, no peer-reviewed studies, to our knowledge, have evaluated Fibrafid supplementation in broilers fed diets with simultaneous reductions in digestible amino acid density and metabolizable energy. Therefore, it remains unclear whether this multifunctional additive can compensate for nutrient dilution while maintaining growth performance, carcass characteristics, meat quality, and intestinal morphology. We hypothesized that Fibrafid supplementation would compensate for reductions in dietary crude protein, digestible amino acids, and metabolizable energy by improving nutrient utilization, thereby maintaining growth performance and carcass characteristics. The 1.5 and 2.5 kg/ton inclusion levels were selected based on the manufacturer’s recommended application range and previous experimental evidence identifying 2.5 kg/ton as an effective level for improving productive performance and intestinal integrity in heat-stressed broilers [48]. Accordingly, this study evaluated the effects of Fibrafid supplementation at these practical inclusion levels in nutrient-reduced broiler diets formulated with lower digestible amino acid density and metabolizable energy relative to current Ross 308 nutritional recommendations.

## 2. Materials and Methods

### 2.1. Ethical Approval

The work was authorized by the Scientific Research Ethics Committee of the Research Council-King Saud University (KSU-SE-21-47) and followed the ARRIVE criteria for animal experiments.

### 2.2. Birds, Management and Housing

The feeding trial was conducted over a 24-day experimental period, during which broiler chickens were reared from 1 to 24 days of age. The overall study extended from 30 July to 4 September 2024, encompassing chick placement, diet preparation, the feeding trial, sample collection at slaughter, and subsequent laboratory analyses of carcass traits, meat quality, and intestinal histomorphology. A floor pen trial was conducted at the Educational and Research Farm in Al-Amariya, KSU, Riyadh, Saudi Arabia. A total of 432 one-day-old Ross 308 broiler chicks (initial body weight: 44.9 ± 0.30 g), obtained from a local hatchery (Al Khumasia Company, Riyadh, Saudi Arabia), were randomly allocated to 36 floor pens (1 m^2^ each), with 12 birds per pen (stocking density: 12 birds/m^2^). Chicks were distributed so that the mean initial body weight was balanced among pens before treatment allocation. The 36 pens were then randomly assigned to the six experimental treatments (six replicate pens per treatment) using a completely randomized 2 × 3 factorial design. Pen locations within the experimental house were also randomized to minimize potential positional effects associated with the housing environment. The temperature was maintained at 34 °C on day 0 and gradually reduced to 22 °C by day 24. Light photoperiod was 23 h light/1 h dark. Relative humidity (RH) was around 50 to 60%. Feed and water were provided ad libitum throughout the trial. Broilers were vaccinated against infectious bronchitis, Newcastle disease, and infectious bursal disease at day 1 via nasal instillation (Fort Dodge Animal Health, Fort Dodge, IA, USA).

### 2.3. Dietary Treatments and Experimental Design

The study followed a completely randomized 2 × 3 factorial design, comprising two dietary nutrient densities (standard and nutrient-reduced diets) and three Fibrafid inclusion levels (0, 1.5, and 2.5 kg/ton). A total of 432 Ross 308 broilers were reared from 0 to 24 days of age and randomly allocated to six treatment groups, each with six replicate pens of 12 birds (6 males and 6 females per pen). The experimental diets differed in metabolizable energy (ME) and digestible amino acid (dAA) density and were fed either without or with Fibrafid supplementation. Accordingly, the six dietary treatments were as follows:

1. PC: Positive control diet meeting Ross 308 nutritional requirements.
2. PC + 1.5 kg/ton Fibrafid: PC supplemented with 1.5 kg/ton Fibrafid.
3. PC + 2.5 kg/ton Fibrafid: PC supplemented with 2.5 kg/ton Fibrafid.
4. NC: Negative control diet with reduced dietary AAs density by 5% and density ME by 1.5%.
5. NC + 1.5 kg/ton Fibrafid: NC supplemented with 1.5 kg/ton Fibrafid.
6. NC + 2.5 kg/ton Fibrafid: NC supplemented with 2.5 kg/ton Fibrafid.

The PC diets were formulated according to the Ross 308 Broiler Nutrition Specifications (2022). The NC diets were formulated by reducing the standardized ileal digestible amino acid (SID AA) density by approximately 5% and ME by approximately 1.5% relative to the PC diets while maintaining the recommended ideal amino acid ratios. Accordingly, all major digestible amino acids, including lysine, methionine, methionine + cystine, threonine, tryptophan, arginine, and valine, were proportionally reduced. As a consequence of the reformulation, crude protein was also moderately reduced (starter: 23.1 vs. 22.4%; grower: 21.5 vs. 20.6%). The calculated ME values were reduced from 2975 to 2930 kcal/kg during the starter phase and from 3050 to 3004 kcal/kg during the grower phase. The ingredient composition and calculated nutrient contents of all experimental diets are presented in Table 1. Dietary formulations were prepared in mash form for the Starter (0–9 days) and Grower (10–24 days) phases (Table 1). Ingredient composition and calculated nutrient contents are expressed on an air-dried (as-fed) basis. Fibrafid supplement was incorporated into feeds as per the manufacturer’s guidelines. Fibrafid^®^ is a plant-derived natural product created by Swiss company Phytaxis SA. It comprises 83% Sweetcane hydrolyzed fiber and wheat straw, 6% glycerol, 5% citric acid, 5% lactic acid, and 1% benzoic acid). The Fibrafid inclusion levels (1.5 and 2.5 kg/ton) were selected based on the manufacturer’s recommended application range and a previous experimental study evaluating Fibrafid supplementation in broiler chickens under heat stress, which identified 2.5 kg/ton as the most effective inclusion level for improving productive performance and intestinal integrity under heat stress [48]. Therefore, 1.5 kg/ton and 2.5 kg/ton were selected to evaluate moderate and higher supplementation levels under nutrient-reduced dietary conditions.

### 2.4. Measurements

#### 2.4.1. Growth Performance and Production Efficiency

Average body weight (ABW) and feed consumption were recorded in each pen at the beginning and the end of each feeding phase. Body weight gain (BWG), feed intake (FI), and feed conversion ratio (FCR, FI/BWG) were calculated on a pen basis. Mortality was recorded and used to adjust performance calculations where applicable. Mortality-corrected FCR was calculated using the traditional standard scientific method [49]. Briefly, FI was adjusted by subtracting the estimated feed consumed by birds that died before the end of the experimental period, with the estimated consumption calculated according to the bird’s age at death. The mortality-corrected FCR was then calculated as adjusted FI divided by the total live body weight gain of the remaining birds. However, no mortality was observed throughout the experimental period.

European Production Efficiency Factor (EPEF) was calculated at the end of the experimental period to assess overall production efficiency using the formula: EPEF = [(Livability (%) × Body Weight (kg))/(Age (days) × FCR)] × 100. Higher EPEF values indicate superior productive performance by integrating growth rate, feed efficiency, survival rate, and market age into a single performance index.

#### 2.4.2. Carcass Characteristics

At day 24, one bird per replicate (*n* = 6/treatment) representing the average pen weight was randomly selected, fasted for 10 h, weighted slaughtered, bled, skinned, eviscerated and the surviving carcass dissected. Birds were slaughtered according to halal guidelines approved by the Institutional Animal Care and Use Committee (IACUC) of King Saud University, ensuring complete exsanguination through a swift neck cut by a trained technician [50].

For carcass characteristics, meat quality, and intestinal histomorphology, one bird per replicate pen (*n* = 6 birds per treatment) with body weight close to the pen mean was randomly selected for sampling. The pen was considered the experimental unit, and the sampled birds were used as representative observations for postmortem and tissue measurements [51,52,53].

Carcass composition and yields were calculated as percentages of live weight, including breast meat, leg weight, abdominal fat, other internal organs, and lymphoid organs following standardized methods [54].

#### 2.4.3. Breast Meat Physicochemical Properties and Quality

To analyze the meat quality later, the left pectoralis major of each broiler was dissected (adipose and connective tissues removed) and stored at −80 °C. To determine the quality of the meat, a number of parameters were evaluated, such as pH, internal temperature, color, water-holding capacity (WHC%), drip loss (DL%), cooking loss (CL%), and the myofibril fragmentation index (MFI) of the breast muscles.

The initial and final physicochemical properties (pH, internal temperature, color) of a freshly cut surface of each breast sample at two different locations were recorded at 20 min and 24 h postmortem (PM) (during this 24 h breast meat was stored at 4 °C).

A portable thermocouple (EcoScan Series, Temp JKT; Eutech Instruments, Singapore) was used to record the internal temperature of the breast muscle. A pH meter (Model pH 211; Hanna Instruments, Woonsocket, RI, USA) was used to record the pH values of the breast muscle. And a CR-400 Chroma Meter (Konica Minolta, Tokyo, Japan) was used to record the CIELAB color system of the breast muscle. The CIELAB color system include coordinates for lightness (L*), redness (a*), and yellowness (b*). To determine color derivatives like the browning index and brightness index, the color data employed a related equation as described by Qaid et al. [55].

Frozen breast muscles were thawed overnight at 4 °C before further meat quality analysis. To assessment WHC%: it was assessed following Wilhelm et al. [56] methodology. According to Suliman et al. [57], the CL% was estimated as the percentage of weight lost during cooking on a typical tabletop grill until the internal temperature of the cooked meat samples reached 70 °C. CL (%) = [(initial weight-final weight)/initial weight] × 100. DL% was determined using the methodology outlined by Pu et al. [58]. Briefly, a small piece of meat was weighted, suspended by paper clips in the box that was covered by the plastic wrap, and stored at 4 °C. The breast meat samples were dried with filter paper and weighted at 24 h postmortem. DL 24 h (%) = [(initial weight − weight 24 h)/initial weight] × 100.

To determined MFI as an indirect indication of calpain activity, 4-g muscle sample was minced by scissors and homogenized by Ultra Turrax (IKA-Werke, Staufen, Germany) with 40 mL of cold MFI buffer. Absorbance of the solution (a 0.5 mg/mL concentration) was measured at 540 nm using a HACH DR/3000 Spectrophotometer (Loveland, CO, USA). MFI was calculated by multiplying absorbance (A 540) by the dilution factor according to the formula: MFI = A_540_ × 200 as described by Suliman et al. [57].

#### 2.4.4. Jejunal Histomorphometry

For histomorphometry analysis, 2 cm segments from the lower jejunum of each slaughtered bird were collected, rinsed with phosphate-buffered saline (PBS), and fixed in 10% buffered formalin. Tissue sections (5 µm thick) of each sample were prepared using a sliding microtome (Leica, SM2010R; Leica Microsystems, Wetzlar, Germany), mounted on slides at 50 °C in a water bath, stained with eosin and hematoxylin [59,60]. To assess intestinal health, villus width and height, along with crypt depth, were measured under a light microscope (Nikon, Eclipse Ni–U microscope, Tokyo, Japan) at 40x magnification. Image analysis was conducted using TS-View (Version 7.1, Tucsen Photonics, China), ImageJ (Version 1.54t; U.S. National Institutes of Health, Bethesda, MD, USA), a calibrated micrometer, an Olympus CX21 microscope, and a digital microscope camera to evaluate intestinal morphology. Villus surface area (VSA) was calculated as following formula: VSA = π × villus width × villus height [49]. Villus height to crypt depth ratio (Villus height/crypt depth) was also calculated.

#### 2.4.5. Statistical Analysis

Data were analyzed using the general linear model (GLM) procedure of SAS software (version 9.4, SAS Institute Inc., Cary, NC, USA; SAS) [61].

The experimental design followed a 2 × 3 factorial arrangement, with dietary regimen (standard vs. nutrient-reduced) and Fibrafid inclusion level (0, 1.5, and 2.5 kg/ton) as fixed effects. The statistical model included the main effects of diet and Fibrafid level, as well as their interaction (Diet × Fibrafid) as follows:

Yijk = μ + D*_i_* + F*_j_* + (D × F)*_ij_* + ε_ijk_

where Yijk is the observed response variable, μ is the overall mean, D*_i_* is the fixed effect of the ith diet, Fj is the fixed effect of the jth Fibrafid level, (D × F)*_ij_* is the interaction effect between diet and Fibrafid, and ε_ijk_ is the residual error.

For growth performance parameters, the pen was considered the experimental unit (*n* = 6 replicate pens per treatment). For carcass characteristics, meat quality, and intestinal morphology, one representative bird was randomly selected from each replicate pen. Consequently, each observation originated from a different experimental pen, ensuring that sampled birds represented independent experimental units and avoiding pseudoreplication. Because one bird was sampled from each independent replicate pen, the sampled birds were not subsamples within a pen but represented independent pen replicates for these endpoint measurements. Prior to analysis, data were checked for normality using the Shapiro–Wilk test and for homogeneity of variance using Levene’s test. When assumptions were met, two-way ANOVA was performed. In cases of significant main or interaction effects, treatment means were separated using Tukey’s HSD test at α = 0.05. Results are presented as the means ± standard error (SEM).

## 3. Results

### 3.1. Growth Performance

The effects of dietary treatment and Fibrafid supplementation on broiler growth performance are presented in Table 2. No significant main effects of diet were observed for ABW, BWG, FI, or FCR during any experimental period (*p* > 0.05).

Fibrafid supplementation influenced body weight at 9 and 24 days of age, BWG during the starter, grower, and overall periods, starter FI, and grower and overall FCR (*p* < 0.05). Birds receiving 2.5 kg/ton Fibrafid showed the highest body weight at 9 days (278 g), 24 days (1293 g), starter BWG (25.9 g/bird/day), grower BWG (72.5 g/bird/day), and overall BWG (52.0 g/bird/day), whereas unsupplemented birds showed the lowest corresponding values. In addition, Fibrafid supplementation improved grower-phase FCR, with lower values observed at 1.5 and 2.5 kg/ton compared with the control group.

Significant Diet × Fibrafid interactions were detected for grower and overall FI (*p* = 0.006 and *p* = 0.023, respectively), while no interactions were observed for body weight, BWG, or FCR (*p* > 0.05). Under the standard diet, supplementation with 2.5 kg/ton Fibrafid resulted in the highest overall FI (63.5 g/bird/day), whereas birds fed the unsupplemented standard diet showed the lowest value (60.4 g/bird/day). In contrast, birds fed the reduced diet without Fibrafid had higher grower FI (89.1 g/bird/day), which declined numerically following Fibrafid inclusion.

Overall, these findings indicate that Fibrafid supplementation improved growth performance and feed efficiency regardless of dietary nutrient density, with the most consistent responses observed at 2.5 kg/ton inclusion.

### 3.2. European Production Efficiency Factor

**Figure 1:**
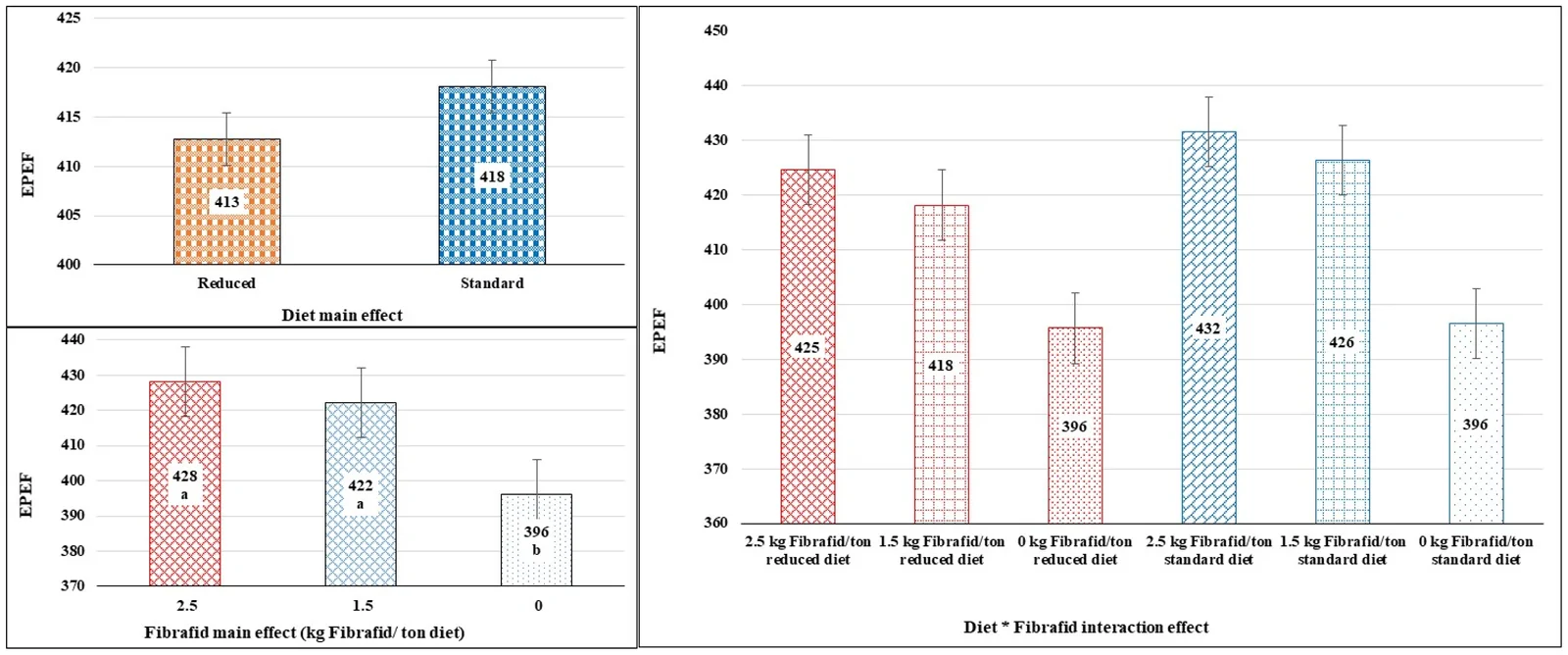
Main effects of dietary nutrient density (standard *vs*. nutrient-reduced diets), Fibrafid supplementation (0, 1.5, and 2.5 kg/ton of diet), and their interaction on the European Production Efficiency Factor (EPEF) of broilers during the experimental period. *p*-values: diet *p* = 0.270; Fibrafid, *p* < 0.0001; diet × Fibrafid, *p* = 0.796.

Figure 1 illustrates the effects of dietary nutrient density and Fibrafid supplementation on the EPEF of broilers. Dietary nutrient density did not affect EPEF (*p* = 0.270), although values were numerically higher with the standard than the nutrient-reduced diet (418 vs. 413). In contrast, Fibrafid supplementation improved EPEF (*p* < 0.0001), with 2.5 kg/ton producing the highest EPEF value (428), statistically similar to 1.5 kg/ton diet (422), and both exceeding the unsupplemented control (396). At the treatment-combination level, 2.5 kg Fibrafid/ton increased EPEF numerically compared with the corresponding controls in both nutrient-reduced (425 vs. 396) and standard (432 vs. 396) diets. Although the diet × Fibrafid interaction was not significant (*p* = 0.796), the highest numerical EPEF was observed with 2.5 kg Fibrafid/ton in the standard diet (432). Overall, Fibrafid improved production efficiency irrespective of dietary nutrient density, with the greatest numerical response at 2.5 kg/ton.

### 3.3. Carcass Features

The impacts of dietary treatment and Fibrafid supplementation on carcass features at day 24 of age are presented in Table 3. No significant Diet × Fibrafid interactions were observed for live weight, carcass weight, dressing yield, breast, leg, wing, neck, proventriculus, heart, liver, or bursa percentages (*p* > 0.05). However, significant interactions were detected for relative weights of gizzard, thymus and abdominal fat deposition (*p* < 0.05).

Fibrafid supplementation at 2.5 kg/ton increased thymus relative weight at day 24 in both dietary regimens, with the highest values recorded in the standard and reduced diets supplemented with 2.5 kg/ton. Conversely, abdominal fat percentage decreased progressively with increasing Fibrafid inclusion, particularly under the reduced diet.

As main effects, reduced diets increased liver and bursa of Fabricius percentages, while decreasing wing proportion and abdominal fat deposition compared with the standard diet (*p* < 0.05). Fibrafid supplementation significantly affected thymus and abdominal fat percentages (*p* < 0.01), with 2.5 kg/ton increasing thymus weight by 41.1% and reducing abdominal fat by 27.6% relative to the unsupplemented control, indicating enhanced lymphoid development and leaner carcass composition.

### 3.4. Breast Physiochemical Characteristics and Meat Quality

The effects of diet and Fibrafid supplementation on physicochemical characteristics of breast meat at 20 min and 24 h postmortem, including pH, core temperature, color components and its derivatives, are presented in Table 4 and Table 5 for 24-day-old broilers.

As shown in Table 4, early postmortem breast meat characteristics showed no significant Diet × Fibrafid interactions at 20 min postmortem (*p* > 0.05). Reduced diets decreased initial pH and increased breast meat temperature, yellowness (b*), and browning index (Bi) of carcasses (*p* < 0.05). Fibrafid supplementation also lowered pH 20 min, increased temperature, and browning index (*p* < 0.01), with no effects on lightness (L*), redness (a*), or whiteness index.

For ultimate meat properties (Table 5), a significant diet × Fibrafid interaction was observed only for carcass temperature at 24 h postmortem (*p* < 0.0001), with the highest value in birds fed the standard diet plus 2.5 kg/ton Fibrafid. However, no significant interactions were observed for pH 24 h or color coordinates (*p* > 0.05). Reduced diets decreased pH 24 h, lightness, and whiteness index, while increasing redness (*p* < 0.05). Fibrafid supplementation reduced pH 24 h and increased core temperature of breast meat irrespective of diet (*p* < 0.01). Overall, broilers fed the nutrient-reduced diet exhibited consistently lower breast meat pH values at both 20 min and 24 h postmortem than those fed the standard diet, regardless of Fibrafid supplementation.

Table 6 shows the effects of treatments on breast meat quality. No significant Diet × Fibrafid interactions or diet main effects were observed for WHC%, DL%, CL%, or MFI (*p* > 0.05). Fibrafid supplementation improved all measured meat quality traits (*p* < 0.05). Increasing Fibrafid levels increased WHC (from 67.3% to 70.6%) and MFI (from 33.0 to 50.3), while reducing DL% and CL% (*p* < 0.05). The greatest improvements were consistently observed at 2.5 kg/ton inclusion.

### 3.5. Jejunal Histomorphometric

The effects of treatments on jejunal morphology are presented in Table 7. Significant Diet × Fibrafid interactions were observed for villus width, villus height, villus surface area (*p* < 0.05), whereas crypt depth and villus height-to-crypt depth ratio were unaffected. Also, a significant Diet × Fibrafid interaction was observed for goblet cell density (GCD, *p* < 0.0001). Overall, GCD increased progressively with increasing Fibrafid supplementation in both dietary treatments, with the greatest values observed at 2.5 kg/ton, although the magnitude of the response differed between the standard and nutrient-reduced diets.

Supplementation with 2.5 kg/ton Fibrafid markedly improved intestinal morphology in both dietary regimens. The highest villus height, villus width and villus surface area were observed in birds fed standard and reduced diets containing 2.5 kg/ton Fibrafid. GCD was also increased by Fibrafid, with maximal values in the standard diet supplemented with 2.5 kg/ton.

As main effects, Fibrafid significantly increased villus width, villus height, villus surface area, and GCD (*p* < 0.05). Reduced diets had no adverse effect on most histomorphometric traits, although a slight decrease in GCD was observed (*p* < 0.001). These findings indicate that Fibrafid improved intestinal absorptive capacity and mucosal integrity irrespective of nutrient density.

## 4. Discussion

The present study evaluated whether Fibrafid supplementation could mitigate the effects of moderate reductions in dietary digestible amino acid density and metabolizable energy during the first 24 days of broiler production. The principal findings demonstrated that although the nutrient-reduced diet alone produced numerical reductions in body weight and feed efficiency, these differences were generally not significant as main effects. In contrast, Fibrafid supplementation, particularly at 2.5 kg/ton, improved final body weight by approximately 5.0%, overall BWG by 5.3%, FCR during the grower period by 4.0%, and the EPEF by 8.08% relative to the control, while also enhancing selected breast meat quality characteristics and jejunal morphology. Moreover, significant Diet × Fibrafid interactions for feed intake and several intestinal morphological traits indicate that the response to Fibrafid depended partly on dietary nutrient density.

The absence of clear responses during the starter phase, followed by more pronounced effects during the grower phase, may reflect age-related development of the gastrointestinal tract rather than an immediate response to supplementation. Young broilers possess an immature gastrointestinal tract and a developing intestinal microbiota, which may limit the early response to dietary interventions [62,63]. As intestinal development progresses, functional feed additives may exert greater effects on digestive physiology and intestinal morphology [59,62,64]. Similar age-dependent responses have been reported for prebiotic- and fiber-based feed additives in broilers, with improvements becoming more apparent after early gut maturation [65,66,67]. However, because intestinal microbiota development was not evaluated in the present study, this explanation should be regarded as a plausible hypothesis rather than a confirmed mechanism.

The improvement in feed efficiency observed with Fibrafid supplementation may reflect complementary effects on gastrointestinal function. Fermentable fiber and associated substrates could have contributed to a more favorable intestinal environment, whereas the organic acid components may have contributed to a more favorable gastrointestinal environment under nutrient-reduced conditions [68,69]. However, because microbial composition, short-chain fatty acid production, digestive enzyme activity, nutrient digestibility, and metabolizable energy utilization were not evaluated in the present study, these mechanisms remain hypothetical. Nevertheless, the present findings are consistent with previous reports indicating that functional feed additives, including phytogenic, prebiotic, and probiotic products, may partially alleviate the performance reductions associated with low-protein or reduced-energy diets [70,71]. Similarly, Strifler et al. [72] reported that performance declined when dietary crude protein and metabolizable energy were reduced unless diets were supplemented with functional additives. This interpretation is also consistent with the nutritional concept proposed by Musigwa et al. [73], who emphasized that maintaining an appropriate balance between essential and non-essential amino acids is fundamental for efficient nutrient use, particularly when dietary crude protein is reduced.

The improvement in EPEF observed with Fibrafid supplementation reflects the combined effects of body weight gain, FCR, viability, and age at marketing rather than a single physiological mechanism [70,74,75]. Accordingly, the higher EPEF values recorded in birds receiving 1.5 and 2.5 kg Fibrafid/ton were primarily attributable to the improvements in body weight gain and FCR during the grower period, indicating enhanced overall production efficiency during the first 24 days of age. Moreover, the absence of marked differences between the standard and nutrient-reduced diets suggests that moderate reductions in dietary nutrient density can be implemented without substantial losses in productive performance when diets are appropriately formulated and supplemented. Although the observed improvements in growth performance and feed efficiency may have practical relevance for commercial production, birds were not reared to market age and no economic evaluation was performed. Therefore, the commercial implications of these findings should be interpreted cautiously and confirmed under full production-cycle conditions.

Most carcass traits were unaffected by either dietary nutrient density or Fibrafid supplementation, indicating that the moderate reductions in metabolizable energy and digestible amino acids were insufficient to alter carcass development during the relatively short experimental period. The absence of significant differences in carcass yield may also reflect the relatively moderate degree of nutrient reduction together with the short experimental duration, which may have limited the expression of treatment effects on carcass development. Likewise, the absence of significant effects on several organ weights suggests that the treatments did not markedly influence overall organ development. In contrast, Fibrafid supplementation reduced abdominal fat deposition, which may reflect altered nutrient partitioning toward lean tissue rather than fat deposition. Although the underlying metabolic mechanisms were not investigated, reduced abdominal fat is generally considered a desirable production outcome because it improves carcass quality and processing yield [76,77].

An increase in thymus weight was also observed in birds receiving nutrient-reduced diets. However, organ weight alone does not provide evidence of improved immune function or reduced inflammatory status. Consequently, these findings should be interpreted cautiously. Previous studies have reported that certain prebiotic and phytogenic additives can influence lymphoid organ development [67,78], but additional measurements, including immune cell populations, cytokine profiles, or humoral immune responses, would be required to determine whether functional immune changes occurred in the present study.

The lower ultimate breast meat pH observed in birds fed the nutrient-reduced diet may be related to differences in postmortem muscle glycolysis. Muscle pH declines as glycogen is converted to lactic acid after slaughter, and dietary nutrient density may influence pre-slaughter muscle glycogen reserves and the rate of postmortem glycolysis [79]. However, because muscle glycogen concentration and glycolytic metabolites were not measured in the present study, this explanation remains speculative and should be interpreted with caution.

Fibrafid supplementation also improved several breast meat quality characteristics, including higher WHC and MFI and lower DL and CL losses. These improvements are important because water retention directly influences processing yield and consumer acceptance [80,81]. The modest increase in ultimate pH and associated changes in meat colour may also have contributed to improved water retention. However, these responses should not be interpreted as evidence of improved meat freshness, since freshness is influenced by numerous postmortem biochemical and microbiological factors that were not evaluated. Likewise, although phytogenic feed additives have previously been associated with improved oxidative stability [82,83], oxidative stress biomarkers were not measured in the present study, and therefore any contribution of reduced oxidative damage remains speculative.

The mechanisms underlying the observed improvements in meat quality are likely multifactorial. Fermentable substrates have been reported to support intestinal fermentation and epithelial development, whereas organic acids can modify the physicochemical conditions within the gastrointestinal tract and improve nutrient digestion. Although these mechanisms have been reported in previous studies [68,69], they were not directly evaluated here and therefore should be regarded only as possible explanations for the observed responses.

Among all measured variables, the intestinal histomorphology findings provide the strongest structural evidence supporting the observed improvements in productive performance. Fibrafid supplementation increased villus dimensions and GCD, with significant Diet × Fibrafid interactions for several morphological traits. Larger villi provide a greater absorptive surface area, while increased GCD may contribute to improved mucus production and epithelial protection [84,85]. Although nutrient digestibility and intestinal permeability were not measured, these structural adaptations may have contributed to improved absorptive capacity and gastrointestinal function. These observations agree with previous reports demonstrating positive effects of prebiotic and phytogenic feed additives on intestinal morphology [59,70,86].

Interestingly, significant Diet × Fibrafid interactions were detected for feed intake and several intestinal morphological variables but not for most carcass characteristics or meat quality traits. This pattern suggests that the influence of Fibrafid was more pronounced for traits directly related to gastrointestinal adaptation than for downstream production characteristics. The absence of interactions for many variables may also indicate that moderate nutrient reduction alone did not substantially challenge physiological homeostasis during the relatively short experimental period.

Several limitations should be considered when interpreting the present findings. First, the feeding trial lasted only 24 days; therefore, responses to market age remain unknown. Second, although diet formulations were based on analyzed ingredient composition, the final nutrient concentrations of the experimental diets were calculated rather than laboratory verified after feed manufacture. Third, no measurements of nutrient digestibility, metabolizable energy utilization, digestive enzyme activity, cecal microbiota, short-chain fatty acid production, intestinal permeability, blood biochemical indices, inflammatory cytokines, or oxidative stress biomarkers were performed. Consequently, the biological mechanisms responsible for the observed improvements remain speculative. In addition, carcass and meat quality evaluations were based on one bird per replicate pen, and no economic assessment was conducted.

Overall, Fibrafid supplementation, particularly at 2.5 kg/ton, was associated with improved growth performance, reduced abdominal fat deposition, enhanced breast meat water-holding capacity, and improved intestinal morphology under both standard and moderately nutrient-reduced dietary conditions. These findings suggest that Fibrafid supplementation was associated with maintaining broiler productivity under the conditions of the present study. Future studies should extend the evaluation to market age and include measurements of nutrient digestibility, cecal microbiota composition, short-chain fatty acid production, intestinal permeability, blood biochemical and inflammatory biomarkers, oxidative stress indices, and economic return to better elucidate the mechanisms underlying the observed responses and determine the practical value of Fibrafid in commercial broiler production.

## 5. Conclusions

In conclusion, dietary Fibrafid supplementation, particularly at 2.5 kg/ton, was associated with improved growth performance, EPEF, reduced abdominal fat deposition, selected breast meat quality traits, and enhanced jejunal morphology in broilers during the first 24 days of age. Under the conditions of the present study, Fibrafid supplementation was also associated with maintaining productive performance in moderately nutrient-reduced diets (5% lower digestible amino acid density and 1.5% lower metabolizable energy). These findings suggest that Fibrafid may serve as a promising natural feed additive for broiler feeding programmes formulated with either standard or moderately reduced nutrient densities. However, because nutrient digestibility, intestinal microbiota, blood biochemical indices, and economic return were not evaluated, further studies extending to market age are warranted to confirm the underlying mechanisms and the practical value of Fibrafid under commercial production conditions.

## Figures and Tables

**Table 1:** Nutritional content and feed components of basal diets (%, as-fed basis).

Ingredients (%)	Starter Period (0–9 d)		Grower Period (10–24 d)	
	Standard Diet “PC”	Reduced Diet “NC”	Standard Diet “PC”	Reduced Diet “NC”
Bakery meal	2.52	2.40	1.90	2.58
Corn	53.7	56.5	58.2	61.5
Soybean meal (48% CP)	36.8	35.2	33.3	30.7
Wheat bran	0.050	0.050	0.000	0.000
Soy oil	2.89	1.80	3.32	2.00
Monocalcium phosphate	1.00	1.00	0.67	0.68
Limestone	1.30	1.30	0.85	0.86
DL-Methionine	0.33	0.38	0.37	0.33
L-Lysine HCL	0.40	0.36	0.32	0.32
L-Threonine	0.15	0.15	0.12	0.11
L-Valine	0.10	0.10	0.07	0.06
Common salt	0.32	0.29	0.32	0.31
Vitamin premix	0.10	0.10	0.10	0.10
Mineral premix	0.10	0.10	0.10	0.10
T-Binder	0.10	0.10	0.10	0.10
Lignobond	0.10	0.10	0.20	0.20
OptiPhos^®^ Plus	0.01	0.01	0.01	0.01
**Total**	**100**	**100**	**100**	**100**
**Calculated composition ^1^**				
Metabolic energy (kcal/kg)	2975	2930	3050	3004
Crude protein (%)	23.1	22.4	21.5	20.6
Calcium (%)	0.95	0.95	0.75	0.75
Available phosphate (%)	0.50	0.50	0.42	0.42
Sodium (%)	0.18	0.18	0.19	0.19
Digestible lysine (%)	1.32	1.25	1.18	1.12
Digestible methionine (%)	0.72	0.67	0.65	0.61
Digestible TSAA ^2^ (%)	1.00	0.95	0.92	0.87
Digestible threonine (%)	0.88	0.84	0.79	0.75
Digestible tryptophan (%)	0.27	0.26	0.25	0.23
Digestible arginine (%)	1.37	1.33	1.27	1.21
Digestible valine (%)	1.00	0.95	0.91	0.86

Abbreviations: PC, positive control (standard nutrient density meeting the requirements of Ross 308 Nutrition Specifications); NC, negative control (reduced-nutrient diet with 5% lower dietary amino acid density and 1.5% lower dietary ME). ^1^ The reported nutrient values are calculated values based on formulation air-dried (as-fed) basis, not laboratory-analyzed values. ^2^ TSAA: digestible total Sulphur amino acids (methionine + cystine).

**Table 2:** Effects of Fibrafid supplementation in standard and nutrient-reduced diets on broiler performance indices during the experimental period.

Diet	Fibrafid (kg/ton)	(ABW g; Age by Day)			BWG (g/bird/day)			FI; g/bird/day)			FCR (Feed/Gain; g/g)		
		0	9	24	Starter	Grower	Overall	Starter	Grower	Overall	Starter	Grower	Overall
Interaction effect													
Standard	0	45.0	271	1237	25.1	69.0	49.7	30.5	84.0 ^b^	60.4 ^b^	1.21	1.22	1.22
Standard	1.5	44.9	275	1279	25.6	71.7	51.4	30.3	87.3 ^ab^	62.3 ^ab^	1.19	1.22	1.21
Standard	2.5	44.9	279	1300	26.0	72.9	52.3	31.7	88.4 ^ab^	63.5 ^a^	1.22	1.21	1.21
Reduced	0	44.9	269	1225	24.9	68.3	49.2	30.3	89.1 ^a^	63.3 ^ab^	1.22	1.30	1.29
Reduced	1.5	44.7	275	1276	25.6	71.5	51.3	30.3	87.2 ^ab^	62.2 ^ab^	1.18	1.22	1.21
Reduced	2.5	44.9	277	1286	25.8	72.1	51.7	31.1	86.4 ^ab^	62.1 ^ab^	1.21	1.20	1.20
Diet main effects													
Standard		44.9	275	1272	25.6	71.2	51.1	30.8	86.59	62.1	1.21	1.22	1.21
Reduced		44.8	274	1262	25.4	70.6	50.7	30.6	87.57	62.7	1.21	1.24	1.24
Fibrafid main effects													
0		45.0	270 ^b^	1231 ^b^	25.0 ^b^	68.6 ^b^	49.4 ^b^	30.4 ^b^	86.5	61.9	1.22	1.26 ^a^	1.23
1.5		44.8	275 ^ab^	1278 ^a^	25.6 ^ab^	71.6 ^a^	51.4 ^a^	30.3 ^b^	87.3	62.3	1.18	1.22 ^ab^	1.21
2.5		44.9	278 ^a^	1293 ^a^	25.9 ^a^	72.5 ^a^	52.0 ^a^	31.4 ^a^	87.4	62.8	1.21	1.21 ^b^	1.21
SEM		0.049	1.203	4.414	0.133	0.279	0.172	0.207	0.478	0.265	0.009	0.006	0.005
Source of variation		*p* value											
Diet		0.392	0.601	0.942	0.629	0.897	0.882	0.864	0.257	0.281	0.621	0.200	0.151
Fibrafid level		0.419	0.020	<0.001	0.018	<0.0001	<0.0001	0.001	0.669	0.122	0.143	0.041	0.098
Diet × Fibrafid		0.954	0.957	0.129	0.956	0.104	0.169	0.757	0.006	0.023	0.874	0.054	0.199

*n* = 6 replicate/group. ^a^ and ^b^ Means in the same columns with different superscript letters indicate significant (*p* < 0.05). Abbreviations: ABW: Average body weight, BWG: daily body weight gain, FI: average daily feed intake, and FCR: feed conversion ratio. Starter (0–9 days), grower (10–24 days), and overall (0–24 days) periods.

**Table 3:** Effects of Fibrafid supplementation in standard and nutrient-reduced diets on broiler carcass characteristics at day 24.

Diet	Fibrafid (kg/ton)	Live Wt. (Kg)	Carcass Wt. (Kg)	DY (%)	Expressed as % from Live Weight											
					Breast	Legs	Wings	Neck	Gizzard	Provent.	Heart	Liver	Bursa	Spleen	Thymus	AF
Interaction effect																
Standard	0	1.293	0.842	64.8	29.4	24.5	4.59	2.12	2.33 ^a^	0.615	0.475	1.78	0.133	0.102	0.247 ^c^	0.499 ^ab^
Standard	1.5	1.322	0.822	62.2	27.5	25.2	5.51	1.75	2.20 ^a^	0.652	0.527	1.99	0.229	0.092	0.350 ^b^	0.451^b^
Standard	2.5	1.353	0.871	64.4	29	25.5	5.19	2.18	1.81 ^b^	0.573	0.52	2.02	0.231	0.105	0.407 ^a^	0.392^c^
Reduced	0	1.26	0.822	65.4	29.4	24.4	4.76	2.26	1.93 ^b^	0.582	0.568	2.10	0.199	0.098	0.335 ^b^	0.545 ^a^
Reduced	1.5	1.297	0.83	64	29.3	25	4.75	1.98	2.62 ^a^	0.615	0.535	2.15	0.276	0.12	0.347 ^b^	0.402 ^b^
Reduced	2.5	1.319	0.854	64.8	29.6	25.2	4.65	2.07	2.27 ^a^	0.498	0.545	2.17	0.288	0.098	0.416 ^a^	0.363 ^c^
Diet main effects																
Standard		1.323	0.845	63.8	28.6	25.1	5.10 ^a^	2.02	2.11	0.613	0.507	1.93 ^b^	0.197 ^b^	0.099	0.335	0.447 ^a^
Reduced		1.292	0.835	64.7	29.5	24.9	4.72 ^b^	2.11	2.27	0.565	0.549	2.14 ^a^	0.254 ^a^	0.106	0.366	0.437 ^b^
Fibrafid main effects																
0		1.277	0.832	65.1	29.4	24.4	4.67	2.19 ^a^	2.13	0.598	0.522	1.94	0.166 ^b^	0.100	0.292 ^b^	0.522 ^a^
1.5		1.31	0.826	63.1	28.4	25.1	5.13	1.87 ^b^	2.41	0.633	0.531	2.07	0.253 ^a^	0.106	0.348 ^ab^	0.427 ^b^
2.5		1.336	0.863	64.6	29.3	25.3	4.92	2.13 ^ab^	2.04	0.536	0.533	2.09	0.258 ^a^	0.102	0.412 ^a^	0.378 ^b^
SEM		0.017	0.013	0.498	0.303	0.178	0.091	0.047	0.083	0.029	0.013	0.040	0.012	0.004	0.015	0.014
Source of variation		*p* value														
Diet		0.390	0.728	0.382	0.175	0.607	0.023	0.297	0.283	0.433	0.110	0.006	0.003	0.387	0.224	0.03
Fibrafid level		0.405	0.519	0.257	0.366	0.131	0.069	0.007	0.132	0.425	0.933	0.185	0.001	0.782	0.002	0.001
Diet × Fibrafid		0.993	0.906	0.843	0.48	0.965	0.052	0.241	0.044	0.953	0.369	0.572	0.909	0.094	0.030	0.006

*n* = 6 samples/group. ^a–c^ Mean values in the same columns with different superscript letters indicate significant (*p* < 0.05). Abbreviations: wt: weight. DY: Dressing yield (%). Provent.: Proventriculus. AF: Abdominal fat.

**Table 4:** Effects of Fibrafid supplementation in standard and nutrient-reduced diets on the physicochemical properties of broiler breast meat at day 24 (20 min postmortem).

Treatment		pH and Core Temperature (CT)		Color Components			Color Derivatives	
Diet	Fibrafid (kg/ton)	pH 20 min	CT 20 min	L*	a*	b*	Bi	Wi
Interaction effect								
Standard	0	6.54	38.4 ^c^	45.2	2.30	9.42	26.8	44.4
Standard	1.5	6.30	39.1 ^bc^	43.2	5.13	10.0	34.7	42.1
Standard	2.5	6.27	40.5 ^a^	45.5	2.62	9.51	27.3	44.5
Reduced	0	6.32	39.5 ^ab^	44.4	3.79	9.54	30.0	43.4
Reduced	1.5	6.26	39.9 ^ab^	43.6	4.52	10.6	35.1	42.4
Reduced	2.5	6.23	40.4 ^a^	44.8	3.93	11.9	37.0	43.3
Diet main effects								
Standard		6.37 ^a^	39.4 ^b^	44.6	3.35	9.65 ^b^	29.6 ^b^	43.7
Reduced		6.27 ^b^	39.9 ^a^	44.3	4.08	10.7 ^a^	34.1 ^a^	43.1
Fibrafid main effects								
0		6.43 ^a^	39.0 ^b^	44.8	3.04	9.48	28.4 ^b^	43.9
1.5		6.28 ^b^	39.5 ^b^	43.4	4.82	10.3	34.9 ^a^	42.2
2.5		6.25 ^b^	40.5 ^a^	45.1	3.28	10.7	32.2 ^ab^	43.9
SEM		0.028	0.174	0.368	0.398	0.234	1.029	0.372
Source of variation		*p* value						
Diet		0.012	0.014	0.552	0.256	0.020	0.008	0.340
Fibrafid level		0.001	<0.0001	0.081	0.059	0.062	0.007	0.053
Diet × Fibrafid		0.089	0.061	0.692	0.335	0.071	0.061	0.586

Abbreviations: Initial color components and its derivatives: L* Lightness; a* redness; b* yellowness. BI: browning index; WI: whiteness index. *n* = 6 samples/group. ^a–c^ Mean values in the same column with different superscript letters indicate significant (*p* < 0.05).

**Table 5:** Effects of Fibrafid supplementation in standard and nutrient-reduced diets on the physicochemical properties of broiler breast meat at day 24 (24 h postmortem).

Treatment		pH and Core Temperature (CT)		Color Components			Color Derivatives	
Diet	Fibrafid (kg/ton)	pH 24 h	CT 24 h	L*	a*	b*	Bi	Wi
Interaction effect								
Standard	0	6.01	15.4 ^c^	48.2	8.35	19.83	68.3	43.1
Standard	1.5	5.83	15.7 ^c^	46.1	7.48	14.8	50.4	43.6
Standard	2.5	5.85	16.9 ^a^	48.7	6.93	14.72	45.9	46.1
Reduced	0	5.82	15.8 ^c^	45.1	8.50	16.83	60.1	41.9
Reduced	1.5	5.75	16.4 ^ab^	43.6	9.73	16.4	63.1	40.4
Reduced	2.5	5.73	16.0 ^ab^	42.0	10.32	17.0	69.1	38.7
Diet main effects								
Standard		5.90 ^a^	16.0	47.7 ^a^	7.59 ^b^	16.46	54.9	44.3 ^a^
Reduced		5.77 ^b^	16.1	43.6 ^b^	9.52 ^a^	16.7	64.1	40.3 ^b^
Fibrafid main effects								
0		5.91 ^a^	15.6 ^b^	46.6	8.43	18.33	64.2	42.5
1.5		5.79 ^b^	16.1 ^a^	44.8	8.61	15.6	56.7	42.0
2.5		5.79 ^b^	16.4 ^a^	45.4	8.63	15.8	57.5	42.4
SEM		0.020	0.122	0.681	0.443	0.881	3.432	0.697
Source of variation		*p* value						
Diet		0.0002	0.813	0.001	0.015	0.859	0.115	0.001
Fibrafid level		0.002	0.001	0.375	0.971	0.270	0.504	0.922
Diet × Fibrafid		0.360	<0.0001	0.242	0.215	0.310	0.088	0.079

Abbreviations: Ultimate color components and its derivatives: L* Lightness; a* redness; b* yellowness. BI: browning index; WI: whiteness index. *n* = 6 samples/group. ^a–c^ Mean values in the same column with different superscript letters indicate significant (*p* < 0.05).

**Table 6:** Effects of Fibrafid supplementation in standard and nutrient-reduced diets on broiler breast meat quality at day 24.

Treatment		Meat Quality Indices			
Diet	Fibrafid (kg/ton)	WHC%	DL%	CL%	MFI
Interaction effect					
Standard	0	67.0	8.48	32.5	34.1
Standard	1.5	68.9	7.02	30.4	41.7
Standard	2.5	70.9	6.81	30.3	50.3
Reduced	0	67.5	8.13	31.4	31.9
Reduced	1.5	69.6	7.45	31.6	41.9
Reduced	2.5	70.3	7.05	30.8	50.2
Diet main effects					
Standard		68.9	7.44	31.1	42.0
Reduced		69.1	7.54	31.3	41.3
Fibrafid main effects					
0		67.3 ^b^	8.31 ^a^	32.0 ^a^	33.0 ^c^
1.5		69.2 ^a^	7.23 ^b^	31.0 ^b^	41.8 ^b^
2.5		70.6 ^a^	6.93 ^b^	30.6 ^b^	50.3 ^a^
SEM		0.377	0.176	0.308	1.811
Source of variation		*p* value			
Diet		0.655	0.678	0.633	0.788
Fibrafid level		<0.0001	<0.0001	0.047	<0.0001
Diet × Fibrafid		0.471	0.446	0.159	0.912

*n* = 6 samples/group. ^a–c^ Mean values in the same column with different superscript letters indicate significant (*p* < 0.05). Abbreviations: WHC%: water-holding capacity, CL%: cooking loss, DL%: drip loss, MFI: myofibril fragmentation index.

**Table 7:** Effects of Fibrafid in standard and nutrient-reduced diets on average histometric indices of jejunum of broilers at day 24.

Treatment		Jejunal Villus Dimensions					Goblet Cell Density (GCD)
Diet	Fibrafid (kg/ton)	Villus Width (μm)	Villus Height (μm)	Villus Surface Area, mm^2^	Crypt Depth (μm)	Villus Height/Crypt Depth	
Standard	0	76.7 ^b^	640.6 ^c^	0.154 ^b^	70	9.13	75.3 ^c^
Standard	1.5	76.0 ^b^	650.6 ^bc^	0.155 ^b^	70.2	9.27	79.3 ^bc^
Standard	2.5	79.8 ^a^	680.5 ^a^	0.171 ^a^	72.2	9.7	98.0 ^a^
Reduced	0	74.0 ^b^	651.3 ^bc^	0.151 ^b^	70.1	9.28	73.0 ^c^
Reduced	1.5	75.8 ^b^	662.6 ^b^	0.158 ^ab^	71.6	9.44	82.3 ^bc^
Reduced	2.5	78.1 ^ab^	680.3 ^a^	0.167 ^a^	72.3	9.69	88.0 ^ab^
Diet main effects							
Standard		77.5 ^a^	657	0.160	70.8	9.37	84.2 ^a^
Reduced		76.0 ^b^	665	0.159	71.3	9.47	81.1 ^b^
Fibrafid main effects							
0		75.4 ^b^	646 ^c^	0.153 ^b^	70.1	9.21	74.2 ^c^
1.5		75.9 ^b^	657 ^b^	0.157 ^ab^	70.9	9.36	80.8 ^b^
2.5		79.0 ^a^	680 ^a^	0.169 ^a^	72.3	9.70	93.0 ^a^
SEM		0.652	3.24	0.002	0.001	0.128	0.306
Source of variation		*p* value					
Diet		0.0002	0.813	0.375	0.105	0.853	<0.001
Fibrafid level		0.001	0.002	0.001	0.907	0.227	<0.001
Diet × Fibrafid		0.020	<0.0001	0.002	0.106	0.916	<0.0001

*n* = 6/group. ^a–c^ Mean values in the same columns with different superscript letters indicate significant (*p* < 0.05).

## Data Availability

The original contributions presented in this study are included in the article. Further inquiries can be directed to the corresponding authors.
